# Diagnosis of cystoisosporiasis in a patient with HIV and modestly decreased CD4 counts

**DOI:** 10.1016/j.idcr.2025.e02259

**Published:** 2025-05-10

**Authors:** Kevin Kurator, Leona Mason, David Bruckner, Hongying Tan, Glenn Mathisen

**Affiliations:** a Olive View-UCLA Medical Center, Department of Medicine, United States; b Olive View-UCLA Medical Center, Department of Infectious Diseases, United States; c UCLA, Department of Pathology, United States; d Olive View-UCLA Medical Center, Department of Pathology, United States

**Keywords:** Cystoisospora, HIV, Biopsy, Stool ova & parasites

## Abstract

Cystoisosporiasis is caused by a coccidian parasite that is found worldwide and can cause diarrheal illness. It is more common in the immunocompromised, in whom it can cause extended and severe disease courses. Diagnosis is not always straightforward as oocytes are not always present in routine stool samples, but it is possible to visualize the organism with specialized staining techniques as well as within enterocytes on intestinal biopsies. We present a case in which this mode of diagnosis was crucial for determining the cause of diarrhea in a patient with HIV and CD4 count above 200 cells/mm^3^.

## Background

**Fig. 1:**
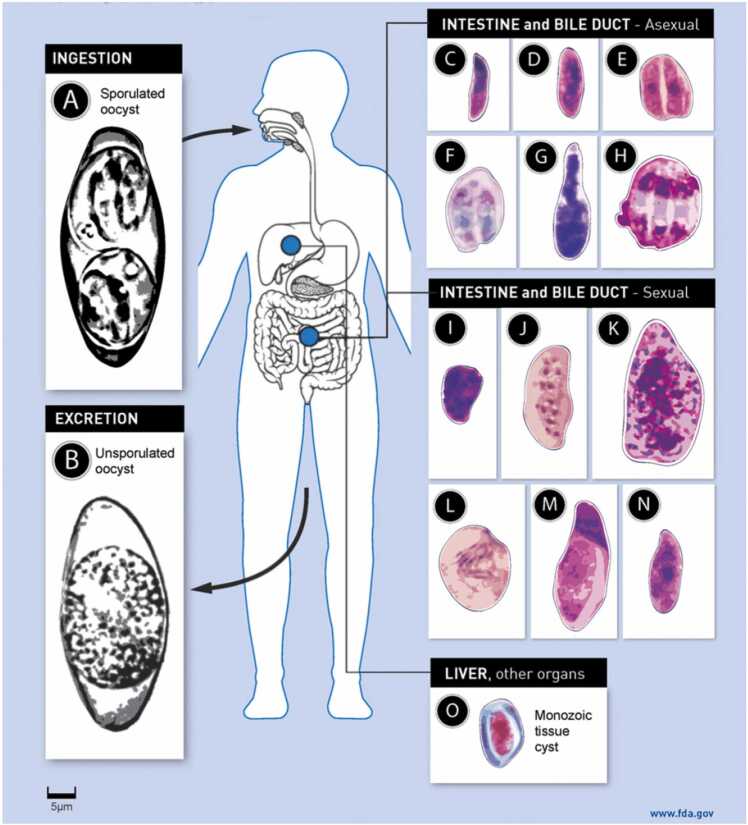
Life cycle of *Cystoisospora belli* (Adapted from Dubey and Almeria [5], p 1512).

*Cystoisospora belli* is an organism known to cause diarrheal illness in humans. Some details about the life cycle and the process of infection remain unclear, but it is understood that oocysts are shed in feces of infected patients and sporulate in the environment to form two sporoblasts, each of which develop into sporocysts containing four sporozoites [1]. Once re-introduced into another host, it is thought that the sporozoites are liberated in the gut lumen where they can infect enterocytes of the intestinal lining. Sporozoites then form merozoites and either reproduce asexually, or in later stages form male and female gametocytes which reproduce sexually, eventually forming new oocysts that are released into the feces (Fig. 1). Oocysts are not consistently shed throughout its life cycle and often not in large quantities, therefore it can be challenging to identify by routine O&P. There are some special stains that may increase diagnostic yield, particularly modified acid-fast staining or safranin staining [2]. Auramine-rhodamine staining has also been explored, with mixed results [3], [4]. In many cases diagnosis is only made by visualization of organisms on an intestinal biopsy [5].

Although infection has been seen worldwide, the distribution of infections is notably heavier in tropical and subtropical regions of the Americas, Africa, Southeast Asia, and the Caribbean [5]. Asymptomatic infection is possible, but common symptoms of cystoisosporiasis include profuse, watery diarrhea, nausea, abdominal discomfort, and in more chronic infections weight loss and malnutrition are possible. It usually has a self-limited course in immunocompetent hosts lasting days to weeks, but chronic and severe infections are more common among the immunocompromised [5]. Spread of infection to the biliary tract has also been reported, and severe infections can lead to profound dehydration and malnutrition.

## Case presentation

**Fig. 2:**
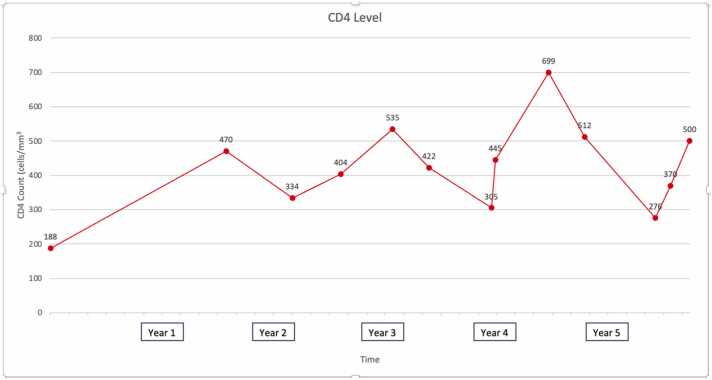
Patient’s CD4 count over time.

The patient is a 49-year-old woman with HIV intermittently on HAART. She was originally from Guatemala and had lived in the US for over ten years. She had presented multiple times over the course of six years to various emergency departments for recurrent episodes of abdominal pain and profuse, non-bloody diarrhea. In the very beginning her CD4 count was 188 cells/mm^3^, but within the first year she had reconstituted to consistently above 250 (and usually above 400, see Fig. 2). Stool testing, including stool culture, O&P, C difficile toxin, cryptosporidium/isospora/microspora testing, and Biofire RT-PCR multiplex gastrointestinal panels including assays for Cyclospora and other parasitic infections were repeatedly negative on multiple occasions. On one occasion she tested positive for C difficile toxin, but showed no improvement in symptoms despite adequate treatment with vancomycin and subsequent negative repeat testing. CT imaging of the abdomen had been done several times with occasional signs of diarrheal illness and enteritis, but otherwise unremarkable. An EGD and colonoscopy in 2018 was unrevealing; biopsy specimens with Warthin-Starry staining in the duodenum, terminal ileum, and rectum showed only mild cryptitis in the ileum. She had also trialed various dietary changes including abstinence from dairy and gluten without effect. During this time her HAART regiment was also changed or self-discontinued multiple times due to concern for an iatrogenic cause; regiments included elvitegravir/cobicistat/emtricitabine/tenofovir, then dolutegravir/emtricitabine/tenofovir, then atazanavir/emtricitabine/tenofovir, then bictegravir/emtricitabine/tenofovir, then dolutegravir/rilpivirine, and finally abacavir/dolutegravir/lamivudine. None of these changes helped her symptoms.

**Fig. 3:**
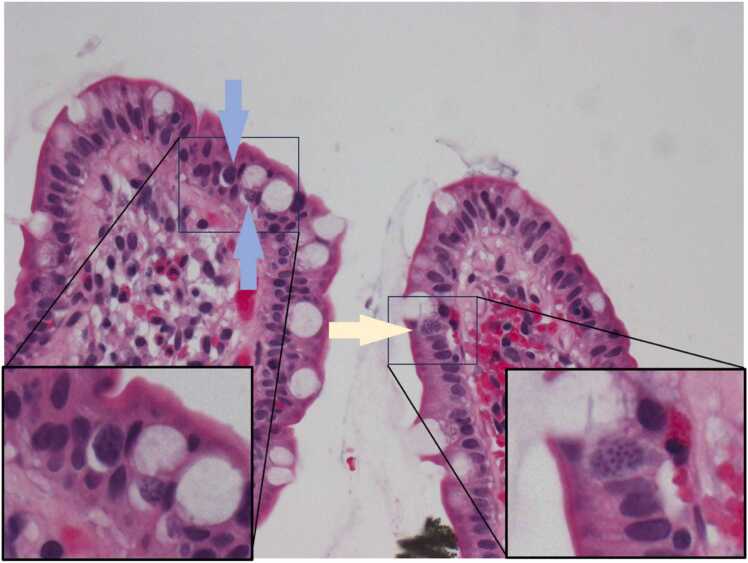
Biopsy taken from terminal ileum showing rare, 10–12 μm intracellular coccidian structures. H&E

**Fig. 4:**
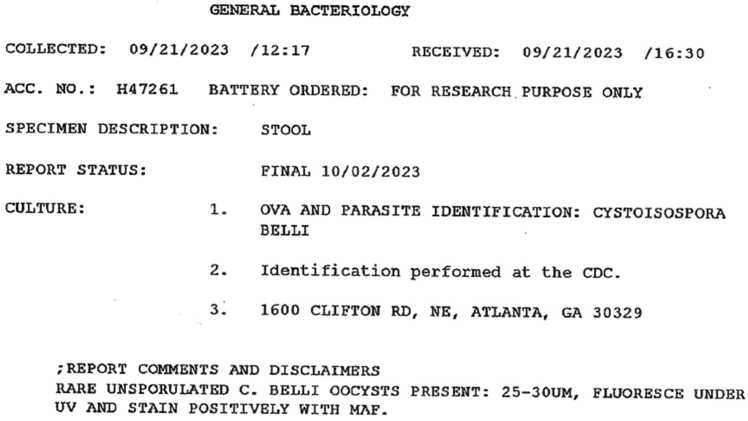
CDC report identifying oocysts in stool sample consistent with *Cystoisospora belli*.

In 2023 she presented again for repeat symptoms of particularly voluminous diarrhea, this time with significant amounts of blood. Repeat stool studies were again negative, and another EGD and colonoscopy was performed with biopsies taken. Pathology results noted focal mild acute cryptitis as well as rare, 10–12 μm intracellular structures within enterocytes that appeared to be coccidian species (Fig. 3). These images and further stool studies were sent to the CDC for testing. The stool samples were examined using fluorescence under UV and modified acid-fast staining and revealed rare, unsporulated 25–30 μm oocysts, identified by the CDC as belonging to *Cystoisospora belli* (Fig. 4). The patient was started on a course of trimethoprim-sulfamethoxazole twice daily with initial improvement of her symptoms, although ultimately had poor adherence and subsequently re-developed diarrhea which is still being treated.

## Discussion

This case highlights the relative unreliability of routine O&P for this particular organism and the utility of more advanced staining techniques, as well as histological evaluation of intestinal biopsy for visualizing intracellular parasites, to confirm diagnosis. Such visualization can be quite subtle. Histological evaluation of the biopsy results in this case shows at least three organisms that appear to be in intracellular stages of the lifecycle, two of which are within parasitophorous vacuoles (yellow and blue arrows). It is difficult to pinpoint precisely which stage they are in, but it is possible they may be in different stages of development which would be consistent with previous reports finding that multiple stages of development can be found within the same infected person [6]. Notably, the larger organism (yellow arrow) is of a similar size as the nuclei of adjacent enterocytes and with a similar degree of staining, and could easily be missed if not examined carefully. This may add to the diagnostic challenge, and it is possible this may have contributed to the delay in identification in this patient who had previously had an unrevealing colonoscopy with biopsy.

Another interesting feature of this case is the fact that the patient was relatively immune-reconstituted with only modestly decreased CD4 counts, and yet was still unable to resolve the infection. This echoes a recent similar case reported in Spain in 2022 in which a young man with well-controlled HIV was also found to have severe diarrhea from cystoisosporiasis that lasted for several years [7]. Although it does seem clear that risk of infection does correlate with CD4 counts [8], the overall impact of patients’ CD4 levels on disease progression or chronicity is not entirely clear, and is complicated by the fact that patients with CD4 counts below 200 are often on prophylaxis for *Pneumocystis jirovecii* and *Toxoplasma gondii* with trimethoprim-sulfamethoxazole, the treatment of choice for *Cystoisospora*. It may be the case that the patient’s initial infection took place because of her initially low CD4 counts, and the persistence of her infection despite improvement in her CD4 counts suggests that immune reconstitution alone is not enough to clear an infection.

Cases like these illustrate the importance of maintaining a broad differential in patients with HIV at any CD4 level and considering the diagnostic limitations of the tests that are employed. They also suggest that patients with HIV may be at elevated risk for disease even at CD4 counts above what is often considered low. This may have significance in considerations of when to start antibiotic prophylaxis in regions that are heavily affected by this parasite. More research is needed to better understand the effect that virologic control and preservation of immune function have on the course of infection with *Cystoisospora belli*.

## CRediT authorship contribution statement

**Mathisen Glenn:** Writing – review & editing, Supervision, Formal analysis, Conceptualization. **Tan Hongying:** Visualization, Formal analysis. **Bruckner David:** Formal analysis. **Mason Leona:** Writing – review & editing. **Kurator Kevin:** Writing – original draft, Data curation.

## Ethical approval

None.

## Consent

Written informed consent was obtained from the patient for publication of this case report and accompanying images. A copy of the written consent is available for review by the Editor-in-Chief of this journal on request.

## Funding

No sources of funding for this publication

## Conflict of Interest

No conflicts of interest to disclose.

## Declaration of Competing Interest

The authors declare that they have no known competing financial interests or personal relationships that could have appeared to influence the work reported in this paper.

## References

[1] Panel on Opportunistic Infections in Adults and Adolescents with HIV. Guidelines for the prevention and treatment of opportunistic infections in adults and adolescents with HIV: recommendations from the Centers for Disease Control and Prevention, the National Institutes of Health, and the HIV Medicine Association of the Infectious Diseases Society of America. p. J-1 to J-6, Available at 〈http://aidsinfo.nih.gov/contentfiles/lvguidelines/adult_oi.pdf〉. [Accessed 4 March 2024].

[2] Key points for laboratory diagnosis of cystoisosporiasis; Cystoisospora beli. DPDx – Laboratory Identification of Parasites of Public Health Concern. 〈https://www.cdc.gov/dpdx/resources/pdf/benchAids/cystoisospora_benchaid.pdf〉. [Accessed 16 March 2024].

[3] Pacheco Flávia T.F., Silva Renata K.N.R., Martins Adson S., Oliveira Ricardo R., Alcântara-Neves Neuza M., Silva Moacir P. (2013). Differences in the detection of *Cryptosporidium* and *Isospora (Cystoisospora)* oocysts according to the fecal concentration or staining method used in a clinical laboratory. J Parasitol.

[4] Abou El-Naga Iman F., Gaafar Maha R. (2014). Auramine-phenol vs. modified Kinyoun’s acid-fast stains for detection of Coccidia Parasites. Lab Med.

[5] Dubey J.P., Almeria S. (2019). Cystoisospora belli infections in humans: the past 100 years. Parasitology.

[6] Rowan D.J., Said S., Schuetz A.N., Pritt B.S. (2020). A case of Cystoisospora (Isospora) belli infection with multiple life stages identified on endoscopic small bowel biopsies. Int J Surg Pathol.

[7] Rial-Crestelo Davida, Otero Bárbarab, Pérez-Ayala Anac, Pinto Adrianaa, Pulido Federicoa (2022). Severe diarrhea by Cystoisospora belli in a well controlled HIV-infected patient. AIDS.

[8] Mohanty I., Panda P., Sahu S., Dash M., Narasimham M.V., Padhi S. (2013). Prevalence of isosporiasis in relation to CD4 cell counts among HIV-infected patients with diarrhea in Odisha, India. Adv Biomed Res.

